# Analysis of Technological Heredity in the Production of Rolling Bearing Rings Made of AISI 52100 Steel Based on Waviness Measurements

**DOI:** 10.3390/ma15113959

**Published:** 2022-06-02

**Authors:** Paweł Zmarzły

**Affiliations:** Department of Mechanical Engineering and Metrology, Kielce University of Technology, al. Tysiąclecia Państwa Polskiego 7, 25-314 Kielce, Poland; pzmarzly@tu.kielce.pl; Tel.: +48-41-342-44-53

**Keywords:** rolling bearings, surface waviness, technological heredity, 100Cr6, AISI 52100 steel

## Abstract

The production of rolling bearings is a complicated process that requires the use of many operations. The manufactured elements of rolling bearings should be of high quality while minimizing production costs. Despite many research studies related to the analysis of technological processes, there is still a lack of research and tools allowing us to satisfactorily assess the relationships between individual operations of the rolling bearing ring process of production and the quality. To perform such an assessment, one can use the concept of technological heredity phenomenon analysis. As the surface waviness of the bearing race is of key importance, the present paper aims at evaluating how the individual technological operations of the rolling bearing ring production process affect the formation of their surface waviness. The surface waviness of the bearing race was measured in both directions (two sections), i.e., along the circumference using the Talyrond 365 measurement system and across the circumference of the race using Talysurf PGI. The production of 6308-2z rolling bearings made of AISI (American Iron and Steel Institute) 52100 bearing steel was analyzed. The occurrence of the phenomenon of technological heredity in the production of rolling bearings was observed. The research results indicate that the turning operation reduces the surface waviness of the bearing rings obtained after forging, while the heat treatment causes a slight increase in surface waviness. On the other hand, grinding operation significantly reduces the waviness, with this reduction being greater for the outer ring. Furthermore, the research has shown that the waviness of the surface is an inheritance factor caused by individual operations of the rolling bearing rings manufacturing process.

## 1. Introduction

Rolling bearings are essential components of technical devices. Rolling bearings in addition to the obvious applications, e.g., in machines and cars [1], are also used in virtually every mechanism that performs a rotary motion. It should be added that the service life of the bearing components depends on the quality of workmanship, which in turn affects the durability and reliability of the mechanisms in which the bearings are installed [2,3]. Conventionally, bearing components are manufactured using plastic forming and machining. Modern technologies such as 3D printing, e.g., SLM technologies, can also be used for the production of rolling bearings [4]. However, due to the limited accuracy of 3D printing technology, it is necessary to use additional operations, e.g., grinding, in order to obtain products with a satisfactory accuracy [5,6].

It may seem that due to the prevalence and standardized character of rolling bearings, the process of their production has been fully analyzed. However, the standardization does not prevent manufacturers of rolling bearings from trying to improve their production processes so that their products are of high quality [7]. In addition, the application of modern materials and additional coatings for the production of bearing elements allows one to improve the functionality of rolling bearings [8]. Moreover, together with the development of methods of manufacturing bearing components, certain solutions related to the measurement technique should be sought in order to improve the measurement process [9,10]. For this purpose, one can use the technological heredity phenomenon analysis in terms of the rolling bearing production process.

In mechanical engineering, in addition to determining the value of deviations in the product, it is important to determine at what stage of the technological process a given deviation (error) occurs and how it is formed during the implementation. Historical information about the magnitude of deviations (errors) from the previous process is important as it enables the determination of how the magnitude of deviations affects the final product. Therefore, the magnitude of the occurring error and its impact on the subsequent part of technological processes should be controlled at each stage of the technological process. We refer to these procedures as the concept of technological inheritance analysis. The term Technological Inheritance (TI) refers to the phenomenon of transferring certain features of an object, e.g., deviation of roundness or waviness of a surface, between successive parts of technological processes. If these properties remain constant in the final product, then the phenomenon of Technological Heredity (TH) occurs.

The technological heredity phenomenon analysis allows us to increase the technological process reliability. This is especially important in the case of complex mechanisms with multi-bolted connection [11]. Thanks to such analysis, it is possible to understand how individual stages of the production process affect the condition of the examined object resulting from, e.g., machining operations. In order to carry out the uncertainty analysis, it is necessary to establish the general principle of heredity for specific parameters, such as shape deviation, waviness, etc. Another concept can be associated with the concept of technological heredity, namely, operational heredity [12,13]. In terms of surface texture metrology and measurement techniques, this phenomenon describes the influence of parameters defining the surface condition on the given component performance properties.

The concept of technological heredity analysis is the subject of several scientific studies. In the study [14], the authors present a concept for the implementation of a scaling strategy into the paradigm of technical inheritance. Markovic and Lazovic in [15] used the phenomenon of technological heredity to evaluate the factors influencing the tribological properties of regenerated gears. Grzesik and others in the paper [16] conducted procedures for the optimization of the hybrid machining process based on the concepts of technological heredity. The optimization aimed to reduce costs and production time, as well as to increase the accuracy of the manufactured components. The authors of the paper presented an interesting approach to the concept of heredity [17], where the evolution of engineering products based on the phenomenon of heredity was presented. The subject of the operational heredity phenomenon assessment is presented in the paper [13], where the influence of the topography of the rolling bearing races on the values of generated vibrations was assessed.

The research presented in the present paper is intended to show how the various stages of rolling bearing ring production affect the formation of surface waviness. The heredity coefficient was used to assess these changes. It should be added that the surface waviness is a critical feature of the rolling bearing race surface [18,19,20]. Choudhury et al. [18] conducted simulations of bearing components including surface waviness analyzed along the circumference of the bearing races. Research indicates that the 2nd waviness component has the highest amplitude. The mathematical model presented in paper [19] confirmed that the surface waviness of the inner bearing ring significantly influenced the values of generated vibrations, which were analyzed in the frequency range 300–1800Hz. Therefore, the value of waviness of the bearing raceway surfaces affects the operational parameters of rolling bearings, such as the values of generated vibrations, noise, and resistance torque [19]. Moreover, surface waviness impacts energy losses in the contact between the inner race and balls and in the contact between the outer race and balls [21]. Sun et al. [22] presented a simulation study that indicated that 3D waviness on the raceway surface has an influence on the bearing vibration and the noise. Waviness deviation of bearing components affected the performance of aerostatic journal bearings [23]. Therefore, the surface waviness should be analyzed in detail. In many scientific studies, the quality of the race surface layer is analyzed by measuring the roughness or waviness of the surface carried out in one cross-section of the measured component. This is a limited approach as the values of these deviations may differ depending on the direction of measurement. In addition, the measurement of the surface topography is carried out only on a certain area of the race, e.g., on the area of 0.9 mm × 0.9 mm. This is too small a measurement area to accurately assess the waviness of the entire race surface. Therefore, the paper analyzes the waviness of the surface measured along the circumference of the race, as well as across the circumference. Such an approach provides more complete information on the race geometry.

## 2. Materials and Methods

### 2.1. Rolling Bearing Rings

For the research on the evaluation of technological heredity, the outer and inner rings of 6308-2z bearings taken directly from the production process were used. Bearings of this type are commonly used in the mechanical industry and can be installed in mechanisms with high rotational speeds. Moreover, they can support axial and radial loads. The rolling bearing rings are made of AISI 52100 bearing steel with the chemical composition and properties given in Table 1.

**Figure 1:**
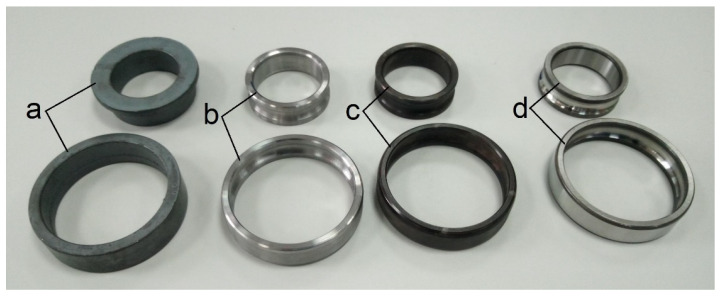
Type 6308-2z rolling bearing rings after successive operations of the technological process: (**a**) rings after forging, (**b**) rings after turning, (**c**) rings after heat treatment, (**d**) rings after grinding.

The production process of type 6308-2z rolling bearing rings includes several key operations. The most important of them were selected to assess the transformation of surface waviness. The first operation consisted of making forgings from a pipe as a blank. Then, the forgings were turned, including turning of the face, as well as the outer and inner surfaces of the ring. Another operation in the manufacturing process of the rolling bearing rings is annealing. The final step is grinding the front, internal, and external ring surfaces. The races are additionally super finished. Figure 1 shows the rings after the subsequent processing stages.

### 2.2. Metrological Measurements

**Figure 2:**
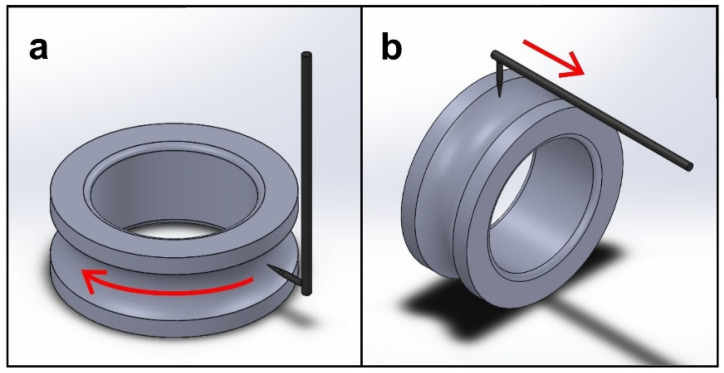
Methods of measuring the waviness of the rolling bearing race surface: (**a**) along the circumference of the race (Talyrond 365), (**b**) across the circumference of the race (Talysurf PGI).

For metrological inspection, three inner and outer rings of 6308-2z bearings taken after specific operations of the technological process were used (Figure 1). In order to fully visualize the transfer of surface waviness of the race as a result of individual stages of the technological process, the measurements of the race were carried out in both directions (two sections), i.e., along the circumference and across the circumference of the race (see Figure 2).

The surface waviness measurements on the circumference of the race were made using a Talyrond 365 roundness measuring instrument manufactured by Taylor Hobson. It is a high-precision measurement system using the radius change method with a rotating table. The parameter used to describe the waviness measured along the circumference of the race is the roundness deviation, RONt, determined based on the least-squares circle, LC. The filtration is performed using a Gaussian filter to separate the roundness and roughness components and leave only the waviness components. This is one of most popular parameters to describe the shape quality of cylindrical elements. It is defined as maximum deviation inside and outside reference circles. As the deviation was determined for the waviness profile, in the remaining part of the paper, the waviness deviation is understood as the RONt parameter.

The measurements of the waviness of the surface race carried out across the circumference of the race were carried out using a Talysurf PGI contact profilometer. Three parameters were used to assess the waviness of the surface race measured transversely, i.e., Wa, Wq, and Wt. Parameter Wa is the arithmetic mean height waviness and is the average of the absolute value along the sampling length. This is one of the most popular waviness parameters applied in industry, but it gives only general information on the waviness without specifying the spatial structure. Another important parameter is root-mean-square waviness deviation described as the Wq parameter. It should be added that the Wa and Wq parameters are used to assess the surfaces that are lubricated and sealed, which is especially important in rolling bearings. Parameter Wt is defined as the total height of the waviness profile along the vertical distance between the maximum profile peak height and the maximum profile valley depth along the evaluation length. The Wt parameter should be used to assess surfaces involved in rotational motion of mechanisms.

Additionally, for selected rings of rolling bearings, the surface topographies were assessed using a Talysurf CCI optical profilometer. Such tests allow for a complete analysis of the surface waviness and the assessment of its change and transfer as a result of individual operations of the bearing ring production process.

### 2.3. The Technological Heredity Factor

To quantify how individual operations of the production process affect the shaping and transmission of surface waviness, the technological heredity factor THF^y^ should be determined (see Equation (1)).

(1) $${\mathrm{THF}}^{y}=\frac{x_{o}-x_{\mathrm{po}}}{x_{o}}\cdot 100\%$$

where *x*_o_—analyzed technological operation parameter value, *x*_po_—previous technological operation parameter value, y—analyzed technological operation index.

The technological heredity factor developed in this way allows determination of the percentage of change and the transfer of the bearing race waviness as a result of individual stages of technological operations in bearing ring production.

## 3. Results and Discussion

**Figure 3:**
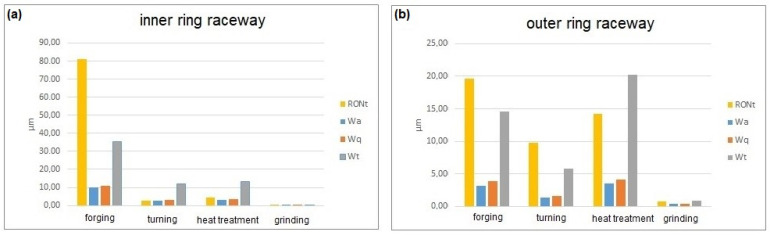
Results of the surface waviness measurements of the inner (**a**) and outer (**b**) races of the 6308-2z roller bearing rings.

The examination effects are presented in graphs and tables, broken down into the results for the races of the inner and outer rings. Figure 3 shows the graphs of the bearing race surface waviness after specific operations of the production process.

In the transfer (inheritance) of the surface waviness parameters between four operations of the rolling bearing ring production process, three heredity coefficients were determined, i.e., THF^t^—technological heredity coefficients for turning, THF^a^—technological heredity coefficients for heat treatment, and THF^g^—technological heredity coefficients for grinding. The values of these coefficients were determined for the waviness deviation RONt and the surface waviness parameters, i.e., Wa, Wq, Wt, and are presented in Table 2 for the inner ring and in Table 3 for the outer ring.

**Figure 4:**
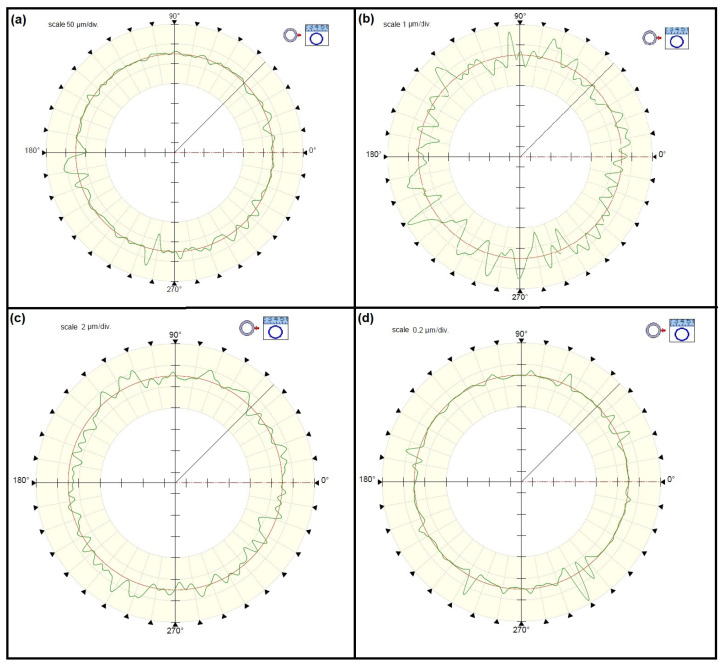
The waviness profiles of the inner ring races after the following stages of machining (**a**) forging, (**b**) turning, (**c**) heat treatment, (**d**) grinding.

**Figure 5:**
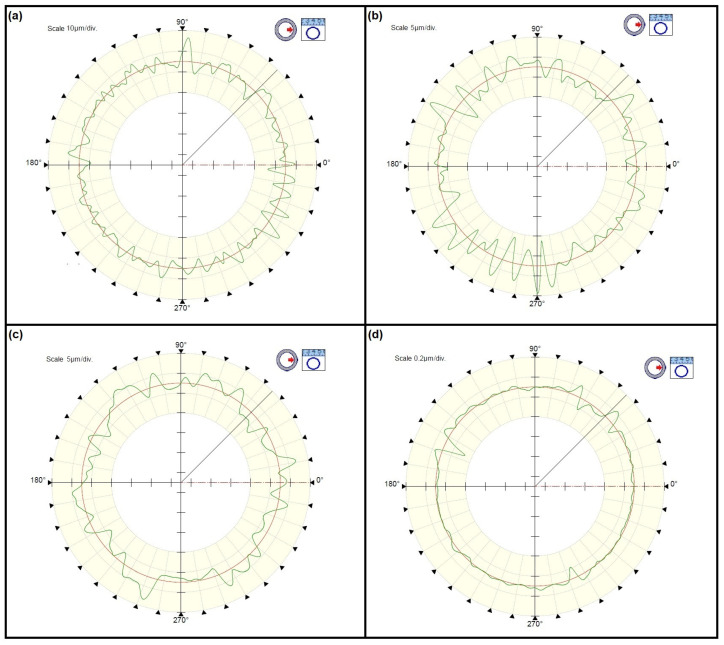
The waviness profiles of the outer ring races after the subsequent machining stages (**a**) forging, (**b**) turning, (**c**) heat treatment, (**d**) grinding.

By analyzing the test results presented in the diagram in Figure 3, it can be concluded that as a result of individual operations of the rolling bearing ring process of production, the surface waviness changes and was inherited between production operations. To visualize the measured deviations, the waviness deviation RONt measured for the races of the inner rings is shown in Figure 4, while Figure 5 shows the waviness deviation RONt for the races of the outer rings.

Considering the waviness deviation RONt measured for the rings of type 6308-2z rolling bearings after forging, it can be definitely stated that the values obtained for the inner ring significantly exceeded the values of the deviations measured for the outer rings (Figure 3). This is due to the forging operation itself, where a characteristic flange remained at the inner rings (see Figure 1), which was then removed. When making a visual assessment of the waviness profiles (Figure 4a), a clear elevation of around 180˚ was visible, proving the occurrence of defects after the forging operation. However, the turning operation caused a significant reduction in the waviness deviation of the inner ring, which was indicated by the technological heredity coefficient of THF^t^ = −3117.22% (Table 2). For the outer ring, this reduction was much smaller and was THF^t^ = −42.96% (Table 3). Similarly, as a result of heat treatment, there were slight changes in the value of the RONt deviation, and the unevenness became more regular (see Figure 4c). As expected, the final grinding treatment significantly reduced the waviness deviation of both the inner (RONt = 0.363 µm) and the outer (RONt = 0.777 µm) races. Compared to the previous technological operation, the deviation decreased by about 12 times. Moreover, the waviness profile became more flat (Figure 4d and Figure 5d).

When analyzing the waviness measured in the direction across the circumference of the race (Figure 2b), one can notice similar trends in the change of the waviness as in the case of measurements along the circumference of the race (Figure 2a). The values of the waviness parameters (Wa, Wq, Wt) were significantly lower than for the waviness deviation RONt. This may result from the high specificity of the measurement and the length of the measurement profile. The parameters Wa, Wq, and Wt were measured only on a short section of the race, while the waviness deviation RONt was determined based on the waviness profile measured along the entire circumference of the race. By analyzing the most popular parameters of the surface waviness, namely, the arithmetical mean height of the waviness profile (Wa) and the root mean square deviation of the waviness profile (Wq), it can be clearly stated that the turning operation reduced the waviness of the inner ring race by about two and a half times and that of the outer ring by about one and a half times. However, as a result of the annealing operation, a slight increase in waviness was observed for both the inner and outer races (see THF^a^ in Table 2 and Table 3). This may result, among other things, from the formation of an oxide layer on the race surface after heat treatment. As for the waviness deviation, RONt, the grinding operation resulted in a significant reduction of the waviness parameters Wa and Wq. For the Wa parameter determined for the inner ring race, the change was the greatest because the technological heredity coefficient for grinding was THF^g^ = −7358.05%.

By analyzing the total height of the waviness profile Wt, one can see that the turning operation reduced the waviness height. As in the case of the Wa and Wq parameters, the heat treatment caused a slight increase in the Wt parameter, while the grinding reduced the inner race waviness to the level of Wt = 0.195 µm and the outer race waviness to the level of Wt = 0.844 µm. It should be added that by definition, the interpretation of the RONt and Wt parameters is similar, as this refers to the distance between the maximum valley and the maximum peak of the waviness profile. Therefore, the correlations between these parameters were high.

**Figure 6:**
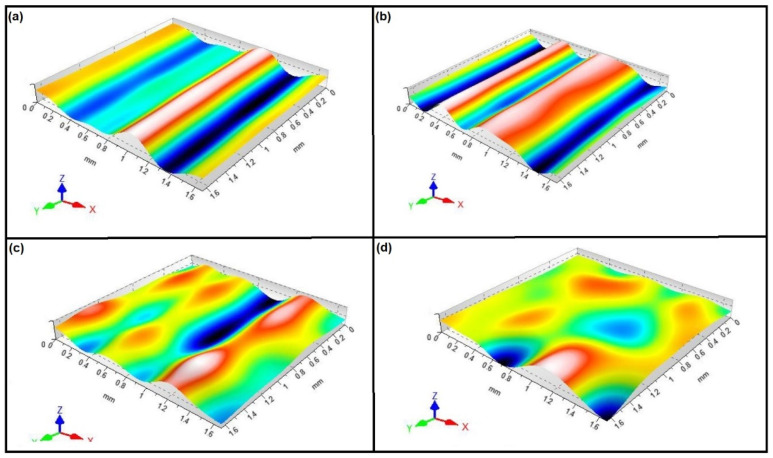
The 3D surface waviness of the inner ring races after the following stages of machining: (**a**) forging, (**b**) turning, (**c**) heat treatment, (**d**) grinding.

The tendencies of an increase or a reduction of surface waviness as a result of individual operations of the manufacturing process for the inner and outer rings were similar. However, these changes were more prominent in the case of the inner ring, which proves the predominant factor of technological heredity. As a result, a more detailed evaluation of the 3D surface waviness was performed. The 3D surface waviness of the inner ring race after the individual machining operations is shown in Figure 6.

Considering the results of the race surface waviness presented in the isometric view, one can notice a different nature of the unevenness after each technological operation. After the forging operation, the surface appeared flatter with only a single dominant height visible, which may be due to errors caused by the forging process (Figure 6a). This was confirmed by the value of the skewness parameter, which for this surface was Ssk = −0.417. The turning operation resulted in the formation of characteristic regular heights, which was the result of mapping the geometry of the cutting insert and the kinematic movement of the machine; also characteristic heights were visible here (Figure 6b). For this surface, skewness was positive (Ssk = 0.261), indicating the surface with great heights. However, after heat treatment (annealing), the waviness of the surface was flatter (Figure 6c). Similar to the surface obtained after forging, the skewness was negative, with indicated materials concentrated around the peak. However, the characteristic turning marks were still visible. By analyzing the surfaces of the inner races after grinding (Figure 6d), one can see that the waviness of the surface in this case was more isotropic. Additionally, there were no such visible machining marks, but the skewness parameter was Ssk = 0.0215 This type of surface is recommended for bearing application. To sum up, it can be concluded that heat treatment and grinding caused the smoothing (flattening of the peaks) of the unevenness represented by the 3D surface waviness.

## 4. Conclusions

The connection of individual operations of the rolling bearing ring process of production with the values of the surface waviness is extremely important because it allows assessment of how the waviness is formed as a result of individual operations and how it affects the final product. It should be noted that waviness deviation affects the operational properties of the bearings, such as the level of generated vibrations. To obtain complete and detailed information that shows the rolling bearings’ race surface waviness, it is necessary to conduct a comprehensive measurement, i.e., measurement along and across the race circumference. In addition, an evaluation of the 3D waviness is recommended.

Research results presented in this paper indicated that individual operations of the technological process of the production of the rolling bearing affected the values of the race surface waviness and the unevenness characteristics. The greatest differences between the surface waviness of the race for the tested outer and inner rings were observed after the forging operation while further operations, i.e., turning and grinding, decreased the surface waviness. However, heat treatment slightly increased the waviness of the raceway surface. Accordingly, this should be taken into account when designing the grinding process to obtain raceways with satisfactory surface waviness. Furthermore, analysis of the surface waviness obtained after the final operation, which was grinding, allowed the conclusion that the surface waviness of the outer ring race exceeded the surface waviness of the inner ring. The developed technological heredity coefficients allow quantifying the number of changes in the waviness of the bearing race surface as a result of individual technological operations. The research showed that the greatest changes occurred for the Wa parameter as a result of the grinding operation.

To sum up, the surface waviness parameters are very important and their value depends on the methods used in the process of production and may affect the functional properties of machine parts. Knowledge of the phenomenon of technological heredity will allow for the appropriate selection of the parameters of individual technological operations to obtain the surface waviness of the raceway with appropriate characteristics so that the bearing meets specific functional properties (low resistance torque, low noise, and low vibration values). Moreover, the values of surface waviness inherited as a result of individual technological operations affect further stages of the process of production. Excessive waviness values also adversely affect subsequent machining operations because they can cause additional vibrations in the entire machine system (machine tool–chuck-cutting tool-piece) and propagation of additional shape errors. This can be eliminated by providing an additional allowance for grinding, but this is very energy-consuming, which is also disadvantageous.

The research presented in the article is preliminary. In further studies, the author will analyze changes in other parameters as a result of other technological operations. The developed technological heredity coefficient will also be used to link the technological parameters of a given manufacturing process with the operational parameters of the manufactured part of the machine. The examination of other types of rolling bearings is planned. Furthermore, in future research, the author will examine changes in the surface texture described by waviness 3D as a result of technological operation parameters.

## Figures and Tables

**Table 1:** The chemical composition and mechanical properties of AISI 52100 steel [24,25].

Chemical Composition						Mechanical Properties in 20 °C			
C	Mn	Si	Cr	P	S	Yield strength	Fracture strength	Young’s Modulus	Poisson’s Ratio
0.99%	0.38%	0.32%	1.45%	0.01%	0.001%	1410 MPa	1867 MPa	201 GPa	0.277

**Table 2:** Technological heredity coefficients determined for the waviness of the inner ring surface.

	THF^t^	THF^a^	THF^g^
RONt	−3117.22%	42.59%	−1106.42%
Wa	−275.17%	10.12%	−7358.05%
Wq	−257.89%	12.48%	−1989.43%
Wt	−197.64%	11.22%	−6765.26%

**Table 3:** Technological heredity coefficients determined for the waviness of the outer ring surface.

	THF^t^	THF^a^	THF^g^
RONt	−42.96%	−32.09%	−1234.76%
Wa	−125.27%	60.86%	−880.33%
Wq	−151.83%	62.46%	−1024.31%
Wt	−154.75%	71.68%	−2299.53%

## Data Availability

Not applicable.
